# 4-aminopyridyl-based lead compounds targeting CYP51 prevent spontaneous parasite relapse in a chronic model and improve cardiac pathology in an acute model of *Trypanosoma cruzi* infection

**DOI:** 10.1371/journal.pntd.0006132

**Published:** 2017-12-27

**Authors:** Claudia Magalhaes Calvet, Jun Yong Choi, Diane Thomas, Brian Suzuki, Ken Hirata, Sharon Lostracco-Johnson, Liliane Batista de Mesquita, Alanderson Nogueira, Marcelo Meuser-Batista, Tatiana Araujo Silva, Jair Lage Siqueira-Neto, William R. Roush, Mirian Claudia de Souza Pereira, James H. McKerrow, Larissa M. Podust

**Affiliations:** 1 Center for Discovery and Innovation in Parasitic Diseases, Skaggs School of Pharmacy and Pharmaceutical Sciences, University of California San Diego, La Jolla, California, United States of America; 2 Cellular Ultra-Structure Laboratory, Oswaldo Cruz Institute (IOC), FIOCRUZ, Rio de Janeiro, Rio de Janeiro, Brazil; 3 Department of Chemistry, Scripps Florida, Jupiter, Florida, United States of America; 4 Department of Pathologic Anatomy, Fernandes Figueira Institute (IFF), FIOCRUZ, Rio de Janeiro, Rio de Janeiro, Brazil; University of Texas at El Paso, UNITED STATES

## Abstract

**Background:**

Chagas disease, caused by the protozoan *Trypanosoma cruzi*, is the leading cause of heart failure in Latin America. The clinical treatment of Chagas disease is limited to two 60 year-old drugs, nifurtimox and benznidazole, that have variable efficacy against different strains of the parasite and may lead to severe side effects. CYP51 is an enzyme in the sterol biosynthesis pathway that has been exploited for the development of therapeutics for fungal and parasitic infections. In a target-based drug discovery program guided by x-ray crystallography, we identified the 4-aminopyridyl-based series of CYP51 inhibitors as being efficacious versus *T*.*cruzi in vitro*; two of the most potent leads, **9** and **12**, have now been evaluated for toxicity and efficacy in mice.

**Methodology/Principal findings:**

Both acute and chronic animal models infected with wild type or transgenic *T*. *cruzi* strains were evaluated. There was no evidence of toxicity in the 28-day dosing study of uninfected animals, as judged by the monitoring of multiple serum and histological parameters. In two acute models of Chagas disease, **9** and **12** drastically reduced parasitemia, increased survival of mice, and prevented liver and heart injury. None of the compounds produced long term sterile cure. In the less severe acute model using the transgenic CL-Brenner strain of *T*.*cruzi*, parasitemia relapsed upon drug withdrawal. In the chronic model, parasitemia fell to a background level and, as evidenced by the bioluminescence detection of *T*. *cruzi* expressing the red-shifted luciferase marker, mice remained negative for 4 weeks after drug withdrawal. Two immunosuppression cycles with cyclophosphamide were required to re-activate the parasites. Although no sterile cure was achieved, the suppression of parasitemia in acutely infected mice resulted in drastically reduced inflammation in the heart.

**Conclusions/Significance:**

The positive outcomes achieved in the absence of sterile cure suggest that the target product profile in anti-Chagasic drug discovery should be revised in favor of safe re-administration of the medication during the lifespan of a Chagas disease patient. A medication that reduces parasite burden may halt or slow progression of cardiomyopathy and therefore improve both life expectancy and quality of life.

## Author summary

Chagas disease is a parasitic disease caused by the *Trypanosoma cruzi*. The infection may result in gastrointestinal manifestations and cardiomyopathy. Benznidazole, the current treatment, has limited efficacy and often leads to serious side effects. Aiming to develop new treatments, our group has identified new inhibitors that block the synthesis of parasitic lipids, resulting in parasite death. In this work, we evaluated the safety and efficacy of two of these compounds, **9** and **12**, in mouse models of *T*. *cruzi* infection. Both compounds were well-tolerated by animals throughout the 28-day administration. In acutely infected mice, the compounds drastically reduced bloodstream parasites and increased survival. When treatment was initiated during the chronic phase, parasitemia dropped to background levels and remained undetectable for 4 weeks after drug withdrawal; parasites were re-activated by chemically-induced immunosuppression. Thus, the experimental compounds tested in these studies had an acceptable safety profile, achieved a marked reduction in parasite load and prevented heart injury due to inflammation, even in the absence of sterile cure. We conclude that the development of non-toxic medications capable of slowing the progression of cardiomyopathy is a valuable treatment option for Chagas disease patients because it could enhance the quality of life.

## Introduction

Chagas disease afflicts about 7 million people in South and Central America [1], where it is the leading cause of heart failure. More than 10,000 deaths are estimated to occur annually due to this disease. Despite joint efforts in Latin America to eradicate the transmission of *Trypanosoma cruzi* through screening of blood banks and control of triatomine vectors, Chagas disease presents a risk to 70 million people living in endemic countries [1,2]. International travel, infected blood transfusions, co-infection with HIV, mother to fetus transmission, and northward migration of the “kissing bug” insect vector [3], all help to drive up the number of cases and push the incidence outside its historic range. Chagas disease is now seen in Europe, North America and Asia and seems set to become an urgent public health issues in countries far beyond its focal source in South America [4,5]. An annual economic burden due to Chagas disease, calculated by simulation models as overall cost, reaches 7.19 billion US dollars, largely from the loss of productivity and premature mortality caused by cardiomyopathy [6,7].

Human infections by *T*. *cruzi* result in a significant mortality rate in children in the acute phase, or may lead to cardiomyopathy in chronically infected adults [8,9]. About 40% of infected individuals develop chronic manifestations of the disease: ten percent of patients develop gastrointestinal symptoms (e.g., mega colon and mega esophagus); and 30% of patients develop cardiac disease characterized by cardiomyopathy, arrhythmias and interstitial fibrosis accompanied by cardiac inflammation[9]. The clinical treatment of Chagas disease is limited to two drugs: nifurtimox and benznidazole, developed about 60 years ago. Nifurtimox is now discontinued in several countries [10,11], while benznidazole has been recently FDA-approved only for use in children of 2 to 12 years old [12,13].

Both benznidazole and nifurtimox are about 80% effective in the acute stage of Chagas disease [14]. Limitations of current therapy include variable efficacy against *T*. *cruzi* of different genetic backgrounds and elevated toxicity with severe side effects, including widespread dermatitis, digestive intolerance, polyneuritis and bone marrow depression, leading to poor patient compliance [10,11]. Both drugs are used in cases of new infections, congenital infections, reactivation and/or re-aggravation associated with immunosuppression and as a preventive measure against laboratory accidents [8]. The efficacy of benznidazole against the more prevalent chronic stage of Chagas dissease was investigated in the BENEFIT clinical trial [15,16]—the first randomized, placebo controlled, clinical study on the effects of benznidazole on the clinical progression of chronic Chagas disease patients with compromised cardiac function. Drug treatment led to a marked reduction of the circulating parasite load in patients from Brazil (strain TcII) and Argentina and Bolivia (strains TcV and TcVI), but not in patients from Colombia or El Salvador (strain TcI). In all cases, benznidazole failed to reduce cardiac function deterioration when evaluated at the 5–7 year follow-up. [15]. These results suggest that benznidazole has limited clinical utility in patients with moderate to advanced cardiac compromise (class I or II heart failure, New York Heart Association terminology). However, an important qualification is that previous observational, not randomized, studies [17] suggest that the drug is effective in patients in the asymptomatic (indeterminate) stage or those with incipient cardiac compromise. A combination of benznidazole with posaconazole in the treatment of asymptomatic patients (the STOP-Chagas clinical trial) also showed no advantage over benznidazole monotherapy, as judged by the PCR test alone. Clinical disease as evidenced by decreased cardiac function or other cardiomyopathy signs were not assessed in this study [18]. In either case, there is an urgent need for safer and more efficacious drugs and drug combinations to meet the etiological challenges of this complex disease.

As an alternative to the use of benznidazole in patients with chronic Chagas disease [19], significant efforts have been made to repurpose antifungal azole drugs targeting sterol biosynthesis. Among validated sterol biosynthetic targets, CYP51 is one of the most extensively exploited for the development of new therapeutics for fungal and parasitic infections [20,21]. The CYP51 inhibitors posaconazole (Noxafil, Merck) and ravuconazole (E1224, Eisai, Tokyo), which have undergone extensive pharmacological and toxicological optimization in antifungal programs, have demonstrated efficacy and curative activity in animal models of Chagas disease [22], and alleviated chronic Chagas disease in a patient with systemic lupus erythematosus [23,24]. Both drugs have been tested in controlled clinical trials for Chagas disease [18,25,26]. The perceived inferiority of both drugs to the current standard-of-care drug, benznidazole, [25,27] was due to their failure to produce sterile cure (PCR negative), and triggered discussions in the Chagas research community about the validity of CYP51 as a target [28,29]. Two concerns have been expressed: (i) differential activity of CYP51 inhibitors against different strains of *T*. *cruzi* or between the replicative (amastigote) and non-replicative (trypomastigote) stages of the parasite and (ii) the slow-acting mechanism of CYP51 versus fast-acting benznidazole [28–30]. A third factor that may have affected the outcomes of the clinical trials is that the repurposed antifungal drugs, including posaconazole and ravuconazole, were not optimized to target *T*. *cruzi* CYP51.

**Fig 1:**
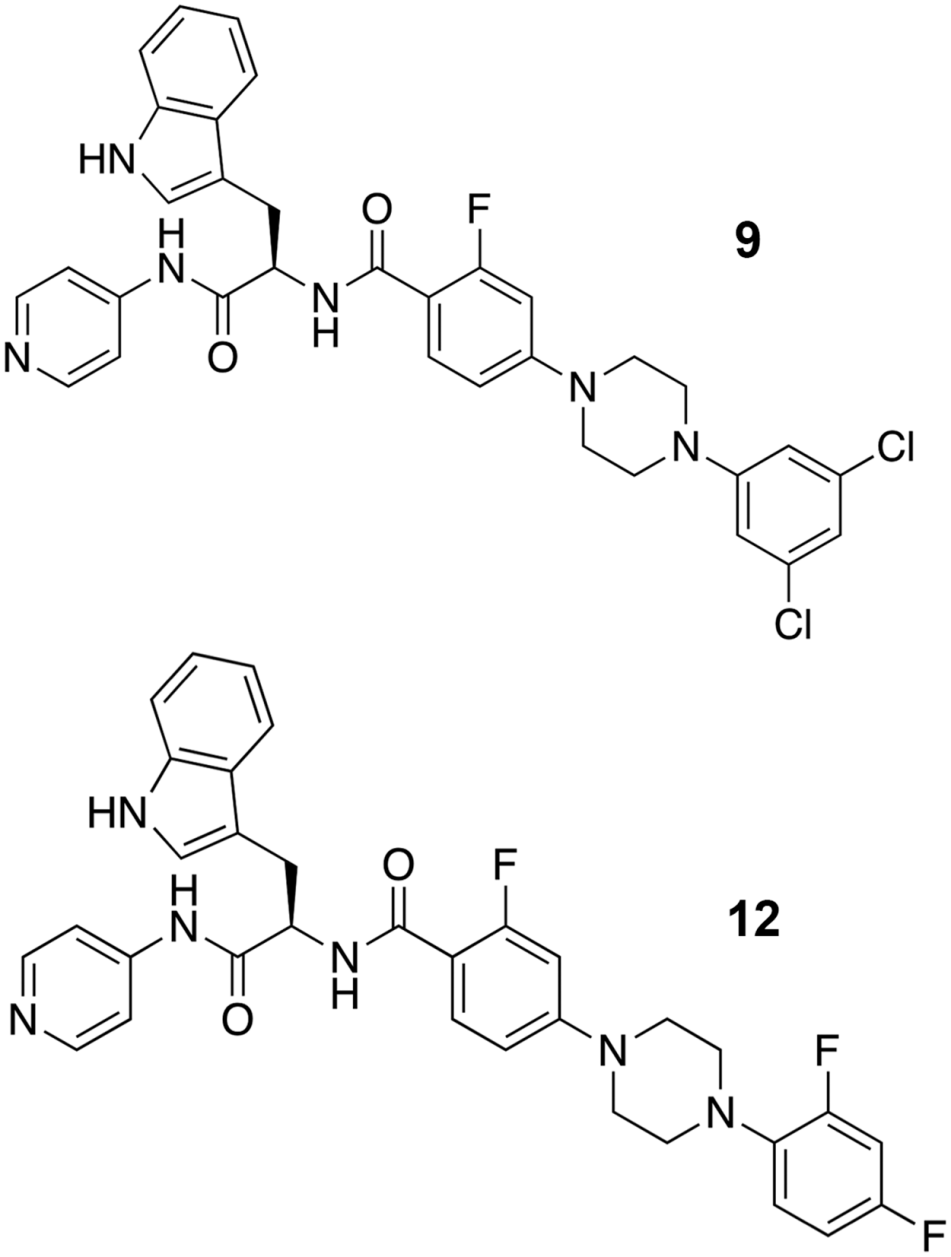
The 4-aminopyridyl-based lead compounds. The lead compounds optimized by the structure-aided rational drug design guided by x-ray crystallography and efficacy in the *in vitro* and *in vivo T*. *cruzi* infection models. Both compounds have EC_50_ in low nanomolar range against cultured amastigotes, are orally bioavailable and decrease 99% parasitemia in mice after 4-day oral dosing [35,37].

In parallel with the clinical trials, a number of laboratories pursued novel chemical scaffolds specifically targeting *T*. *cruzi* CYP51 (reviewed in [21]). Using a target-based structure-aided drug discovery approach, a 4-aminopyridyl-based scaffold was identified as efficacious and further developed into a series of lead compounds active against *T*. *cruzi* both *in vitro* and *in vivo* [31–37] (Fig 1). Two optimized leads of the series, **9** [37] and **12** [35] (Fig 1, compound numbers correspond to those in the cited references), have now been evaluated for both toxicity and parasitological cure in the acute and chronic animal models of *T*. *cruzi* infection. Although a sterile cure was not achieved, **9** and **12** were proven safe for long term administration in mice and suppressed parasitemia in both the acute and chronic phases. In the acute model, these lead compounds improved survival, protected mice from hepatic injury and drastically reduced cardiac inflammation. In the chronic phase, these lead compounds prevented spontaneous *T*. *cruzi* relapse for up to 4 weeks post-treatment.

## Results

### Toxicity evaluation in uninfected mice

**Fig 2:**
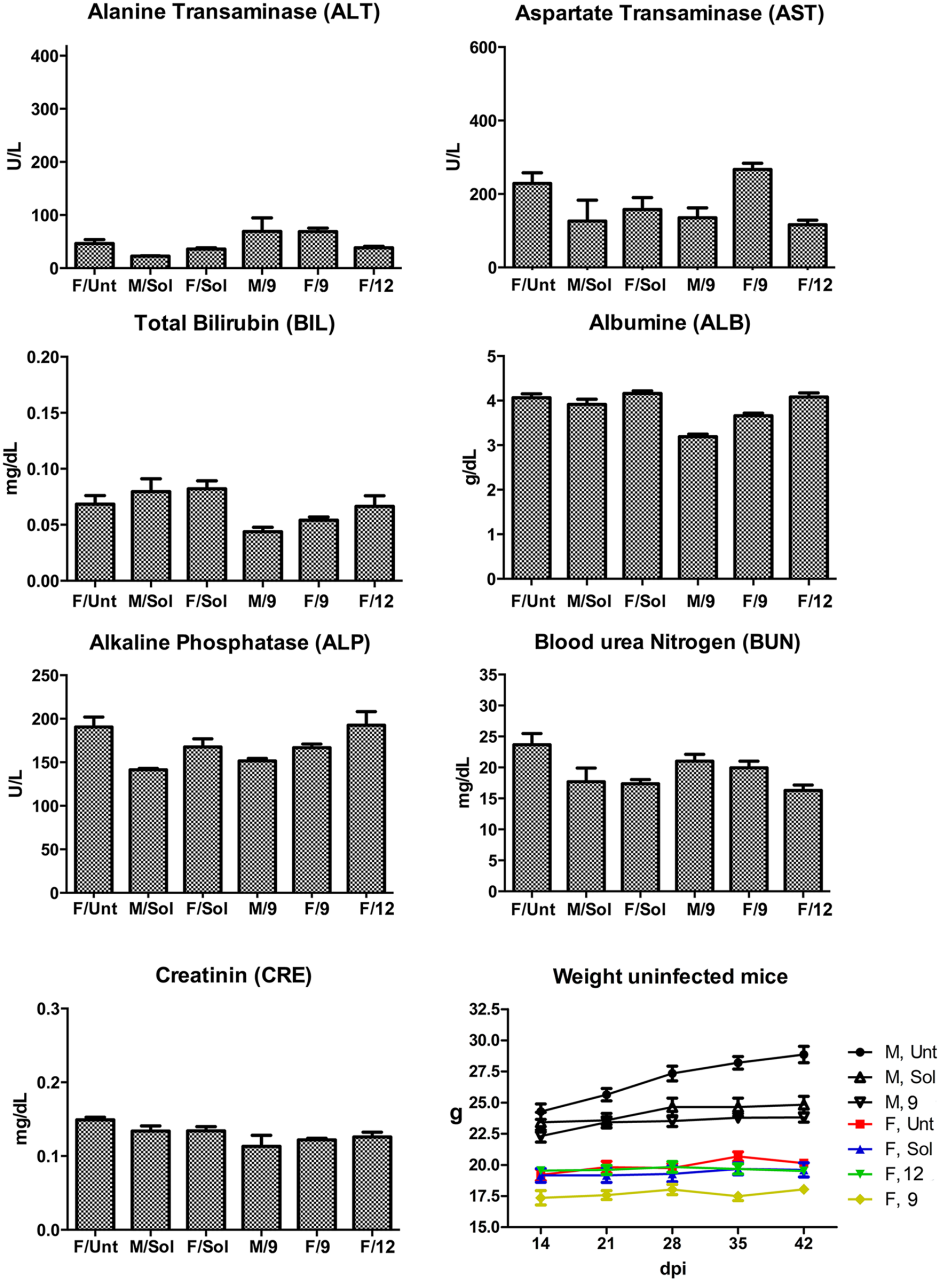
Cumulative *in vivo* effect of 9 and 12. No alteration in the liver (ALT, AST, BIL, ALB, ALP) or kidney (BUN, CRE) markers were observed in the course of mice treated with **9** or **12** at 25 mg/kg, for 28 days, b.i.d. Treatment groups are labeled along the x-axis by the gender/drug combination.

The No Observed Adverse Effect Level (NOAEL), of **9** and **12** was evaluated according to the Organization for Economic Cooperation and Development (OECD) guidelines. Escalating doses of **12** were administered orally to male and female Swiss mice every hour; adverse effects were observed only at concentrations higher than 300 mg/kg for male and 250 mg/kg for female mice. Cumulative *in vivo* effects were analyzed using uninfected BALB/c male and female mice treated with **9** or **12** at 25 mg/kg orally for up to 28 days, b.i.d. No adverse clinical signs (such as ruffled fur, hunched posture, reduced mobility, or tremor) or alteration in general health were observed in any of the mice. Blood was collected after the end of treatment and serum was evaluated in a chemistry panel that included liver enzymes and markers of renal function. No alteration of blood levels for alanine aminotransferase (ALT), aspartate aminotransferase (AST), bilirubin (BIL), albumin (ALB), blood urea nitrogen (BUN) or creatinine (CRE) was detected after the course of treatment (Fig 2). Histological analysis of brain, heart, liver, kidney, GI tract and lungs did not show any alteration of tissue morphology. The weights of the animals remained steady throughout the treatment.

### Effect of compounds on severe acute infections with the lethal *T*. *cruzi* Y strain

**Fig 3:**
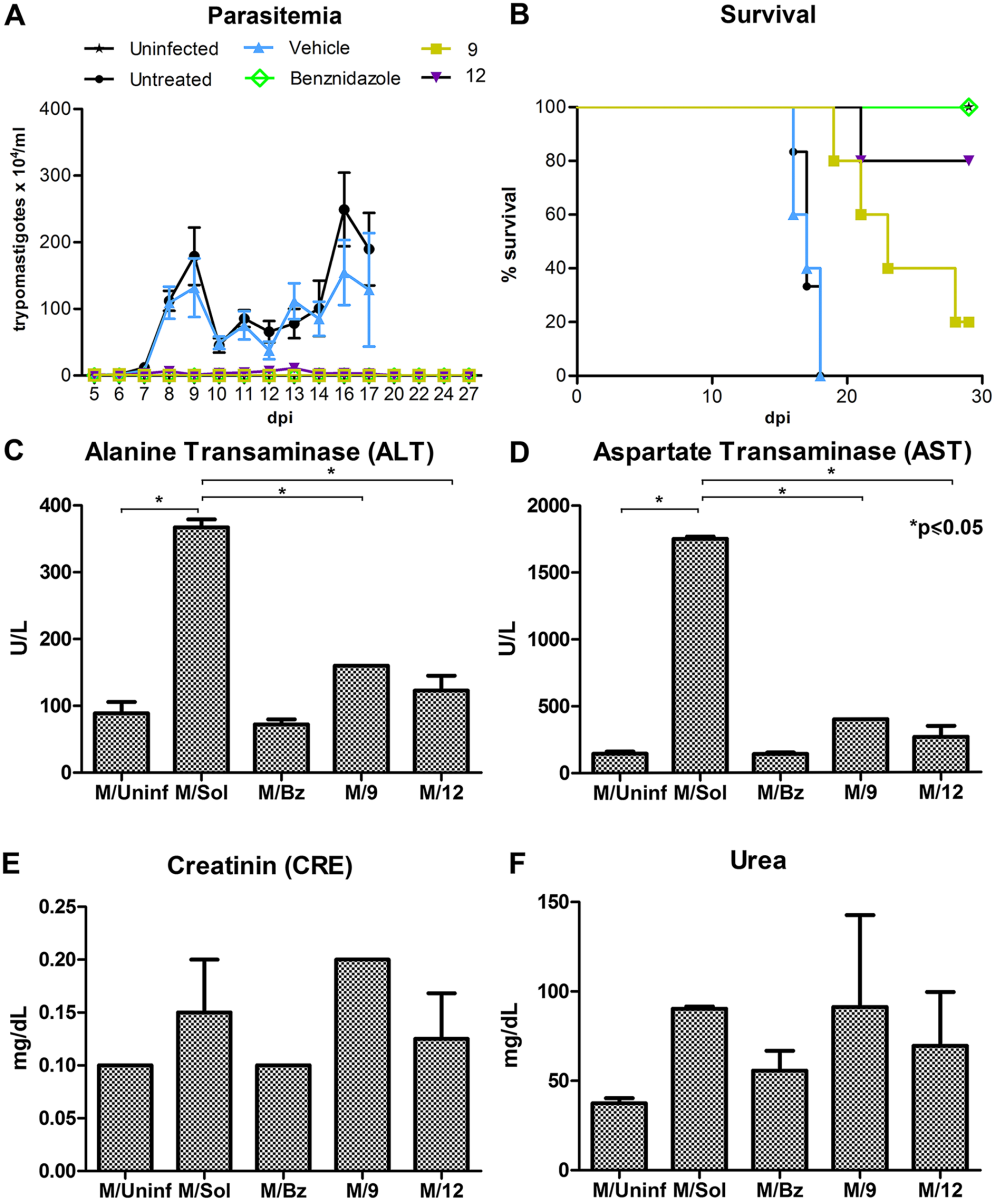
The effects of 4-aminopyridyl lead compounds on mice with acute *T*. *cruzi* infection. (**A**) Parasitemia levels in Swiss male mice infected with *T*. *cruzi* Y strain (10^4^ inoculum) and treated for 28 days with **9** or **12** (25 mg/Kg) are undetectable. (**B**) Survival curve shows partial protection and delayed death of mice treated with **9** and **12** (25 mg/Kg) in the acute phase of the infection. (**C-F**) Biochemical analysis of serum from infected mice treated with **9** or **12** (25 mg/Kg) was comparable to serum from unifected controls and shows normal levels of liver enzymes—alanine aminotransferase (ALT;C) and aspartate aminotransferase (AST;D)–as well as the renal function markers—creatinine (CRE; E) and urea (F). dpi—days post infection. *Statistically significant by *t* test, p≤0.05.

Since no toxicity was detected in uninfected mice, we performed a 28-day oral treatment at 25 mg/kg of compounds in Swiss male mice infected with *T*. *cruzi* Y strain (10^4^ inoculum), an established model of acute infection recommended for drug screening and development by the Fiocruz Program for Research and Technological Development on Chagas Disease (PIDC/Fiocruz) and the Drugs for Neglected Diseases Initiative (DNDi) [38]. Treatment with **9** or **12** at 25 mg/kg significantly reduced parasitemia, reaching the minimum limit of detection by the Pizzi-Brener method at 9 days post infection (dpi), with inhibition levels of 99.9% for **9** and 99.3% for **12**. Parasitemia remained undetectable in the treated mice, while untreated and vehicle-treated mice showed high parasitemia and all of these mice died by 18 dpi (Fig 3A). While parasites were not detected, only 20% of the infected mice treated with **9** and 80% of the infected mice treated with **12** survived the entire 30 day study (Fig 3B). 100% of benznidazole-treated mice survived. All mice were euthanized at the end of treatment. Compared to the negative controls, treatment with the test compounds did provide partial protection and delayed the death of the mice. Death of the treated animals in this model of acute disease with Y strain parasites may be due to yet unknown *T*. *cruzi* Y strain-specific factor(s) that could interfere with the compound effect in the infected mice. That effect with Y strain parasites was not seen in uninfected animals or animals infected with *T*. *cruzi* CL-luc strain.

Upon study completion, mice were euthanized and serum was assessed for hepatic enzymes and renal function markers (Fig 3C–3F). Data from the blood chemistry analysis showed that *T*. *cruzi* Y infection induced elevated serum levels of alanine aminotransferase (ALT) and aspartate aminotransferase (AST), indicating infection-induced liver injury. The animals treated with **9** or **12** had reduced levels of these enzymes when compared to untreated controls. Urea and creatinine (CRE) levels, markers of renal function, were also analyzed; treated animals show normal levels similar to that of untreated controls, suggesting no renal toxicity was caused by **9** or **12**.

### Effect of 9 and 12 on less severe acute infections with the transgenic CL-Brenner *T*. *cruzi* strain in mice

**Fig 4:**
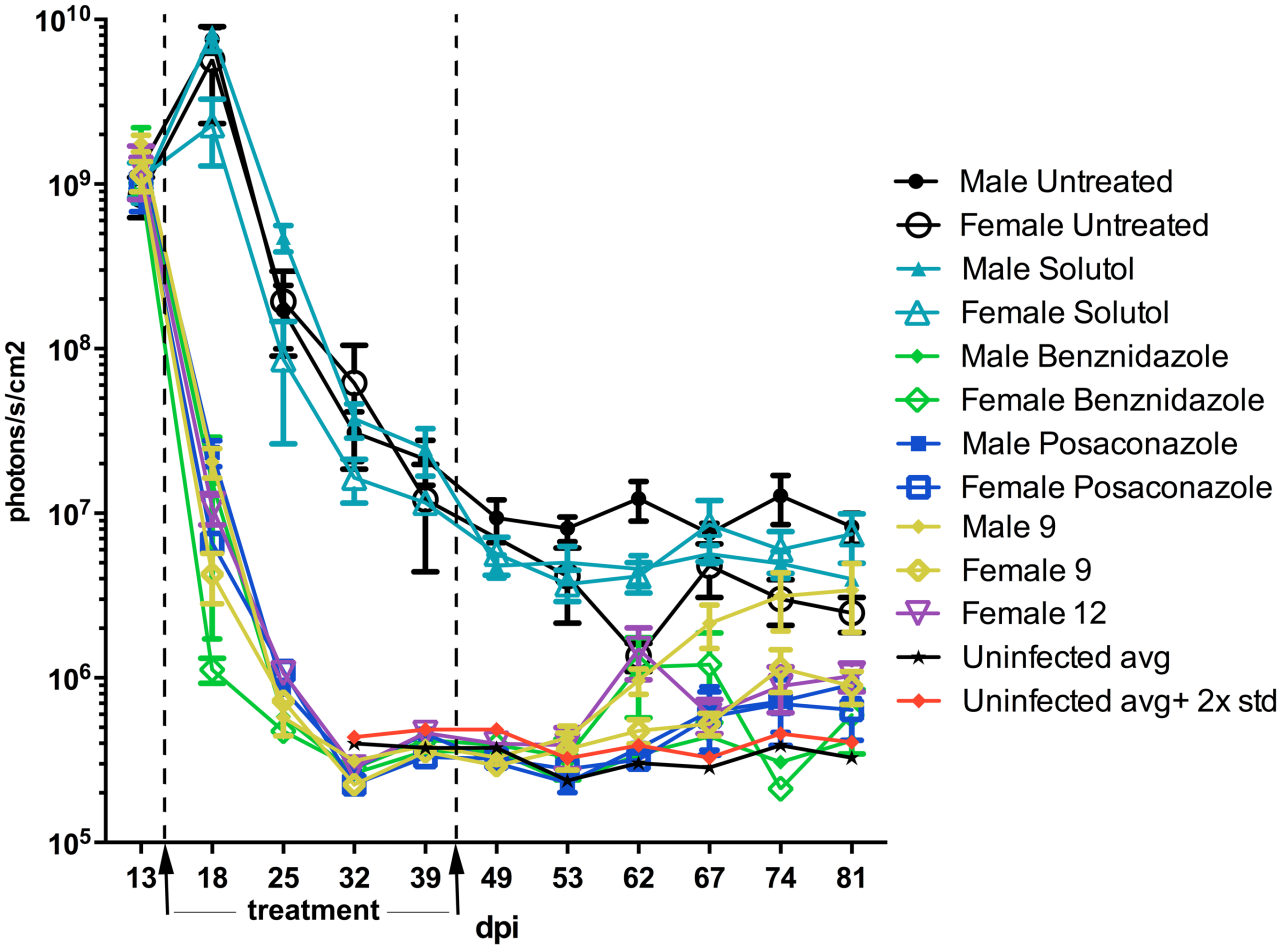
Monitoring parasitemia in acutely infected with *T*. *cruzi* CL-luc BALB/c mice over the course of 28-day treatment with 9 or 12. Bioluminescence levels of infected and treated animals are indistinguishable from those of uninfected control groups between days 28 and 49 post-infection. Parasite relapse ocurred two weeks after the end of treatment, with males eventually reaching bioluminescence levels similar to untreated controls, while females display lower levels of parasitemia.

**Fig 5:**
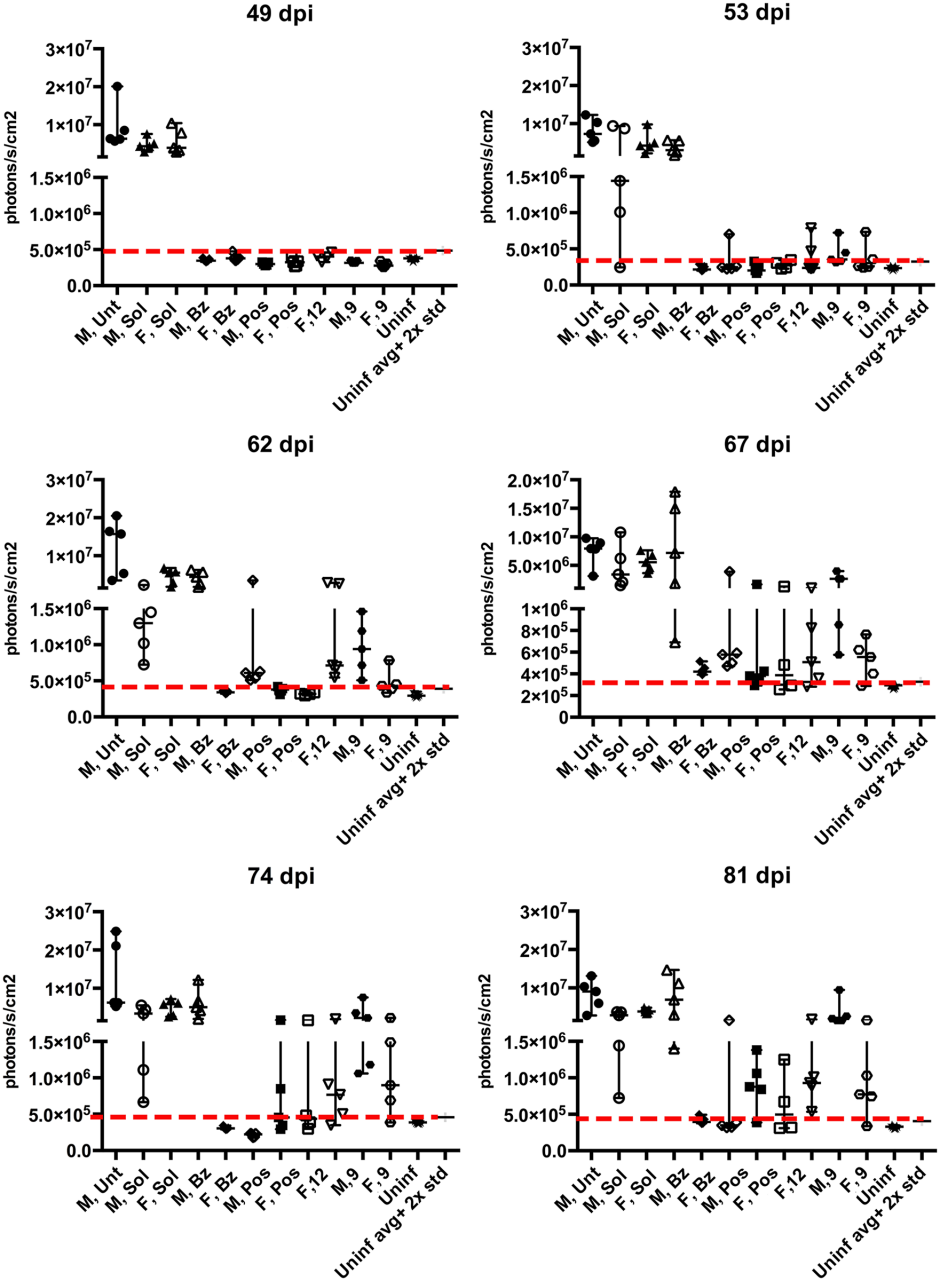
Development of parasitemia in acutely infected mice after the 28-day course of treatment with 9 or 12. The photon count values are displayed for each individual animal up to 81 dpi, showing progressive parasite relapse. Post-treatment evolution of parasetimea in live animals is shown in Fig 6.

**Fig 6:**
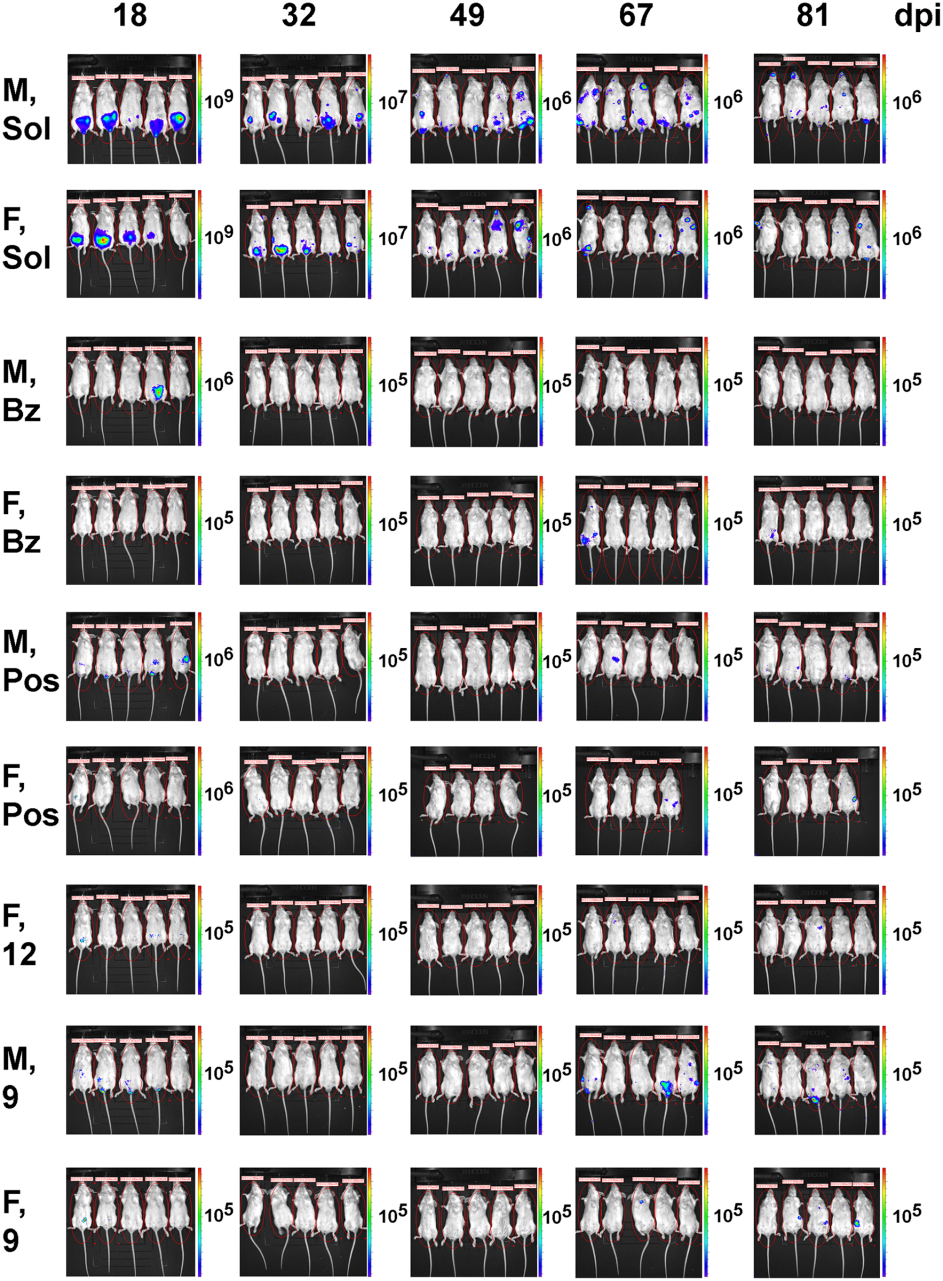
Post-treatment evolution of parasitemia in acutely infected mice monitored by bioluminescence imaging of live animals. Stochastic profile of *T*. *cruzi* infection is shown. Abbreviations are M—male, F—female, Bz—benznidazole, Pos—posaconazole, Sol—solutol, **9** and **12** –lead compounds.

Given that neither **9** nor **12** produced sterile cure in the *T*. *cruzi* Y-infected animals, we next evaluated their effect in a less severe acute model using the transgenic CL-Brenner *T*. *cruzi* strain expressing “red-shifted” luciferase (CL-luc, a gift from Dr. John Kelly, UK). This strain carries a stable bioluminescent marker, which allows one to detect live parasites in tissues of a live mouse with a sensitivity limit exceeding that of the RT-PCR method for up to a year after infection [39,40]. Since mice gender influences the level of infection [41,42], we used both male and female mice for the 28 day b.i.d. treatment beginning 14 dpi. Males not treated with experimental or reference compounds showed levels of parasitemia higher than females (Figs 4, 5 and 6).

As judged by the bioluminescence (Fig 4), the level of CL-luc *T*. *cruzi* infection markedly decreased at 18 dpi, which corresponds to 4 days of treatment with the experimental or reference compounds, benznidazole or posaconazole. By 32 dpi (18 days of treatment), the parasite bioluminescence in all treated mice was reduced to the background level, with the photon count lower than that of the uninfected mice injected with luciferin. Both groups of negative control mice, including a group of infected but untreated mice, as well as a group of infected and vehicle-treated mice, had bioluminescence levels two orders of magnitude higher than the uninfected control mice. This trend was maintained up to 49 dpi (7 days after the end of treatment). However, by 53 dpi (11 days after the end of treatment) animals from the benznidazole (male and female), **12** (female), and **9** (male and female) groups all showed resurgence of parasites (Figs 4, 5 and 6). Posaconazole treated mice showed resurgence of parasites at 67 dpi.

By 81 dpi, the majority of the animals in the **9-** and **12**-treated groups were *T*. *cruzi-*positive as indicated by bioluminescence. All animals in the male group treated with **9** showed parasitemia as high as untreated controls at 81 days post infection. Both compounds reduced the parasite load in female mice, where bioluminescence levels were 10× lower than untreated controls in 4 out of 5 mice for both compounds at 81 dpi, with one **9**-treated female negative (Table 1). Posaconazole reduced *T*. *cruzi* bioluminescence to undetectable levels in 1 out of 5 males and 2 out of 4 females, with the remaining mice showing reactivation of parasites. Posaconazole markedly reduced parasite load compared to the untreated controls. Finally, benznidazole was not able to suppress parasites in all the mice, with one female showing circulating parasites after treatment withdrawal (Figs 4, 5 and 6, Table 1).

**Table 1:** Parasite load on 81 day post-infection by the luminescence level.

Compound/vehicle	Mice sex	Photons/s/cm^2^ ≥10^6^	Photons/s/cm^2^ ≥10^5^	Negative (≤ uninfected)
-/-	Male	5/5	0/5	0/5
-/-	Female	3/5	2/5	0/5
-/solutol	Male	5/5	0/5	0/5
-/solutol	Female	4/5	1/5	0/5
BZ/solutol	Male	0/5	0/5	5/5
BZ/solutol	Female	1/5	0/5	4/5
POS/solutol	Male	0/5	4/5	1/5
POS/solutol	Female	0/4	2/4	2/4
**9**/solutol	Male	5/5	0/5	0/5
**9**/solutol	Female	1/5	3/5	1/5
**12**/solutol	Female	1/5	4/5	0/5

BZ—benznidazole, POS—posaconazole

**Fig 7:**
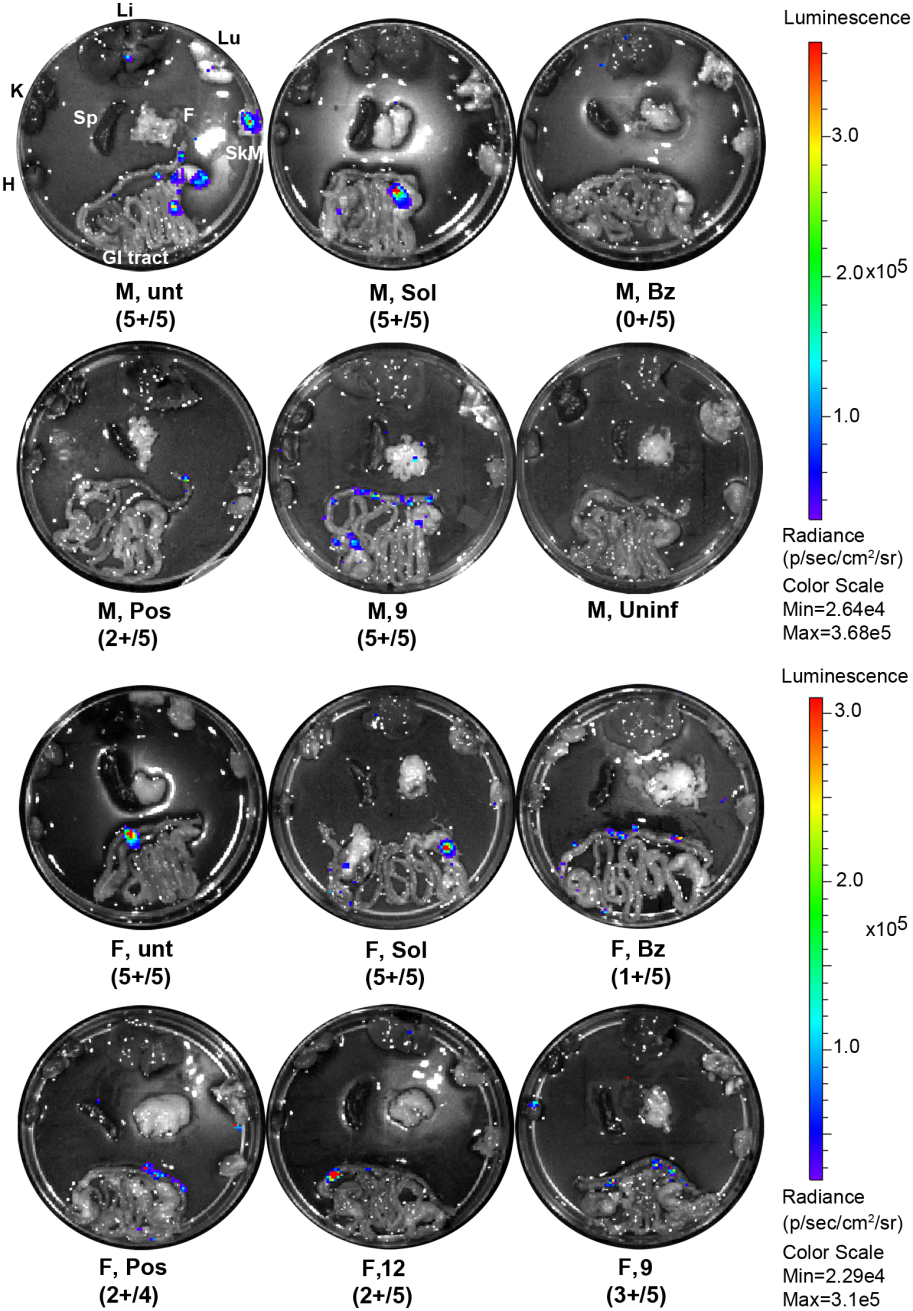
*Ex vivo* bioluminescence imaging of the internal organs of chronically-infected mice. Gastro-intestinal (GI) tract, heart (H), skeletal muscle (SkM), liver (Li), lung (Lu), spleen (Sp) and mesenteric fat (F) are shown uniformly positioned on a petri dish surface as indicated in the top left image. Each group of mice is represented by one image labeled with the animal gender, compound name and a number of *T*. *cruzi*-positive animals in the group of five. Collectively, bioluminescence was detected in the GI of all *T*. *cruzi*-positive animals and randomly in heart, skeletal muscle, liver and mesenteric fat. Animals in the **9**- or **12-**treated groups showed *T*. *cruzi* bioluminescence in the GI tract, liver, lung and mesenteric fat. Bioluminescence was not detected in one female treated with **9** and two females treated with **12**. Quantitative analysis of all animals is shown in Fig 8.

**Fig 8:**
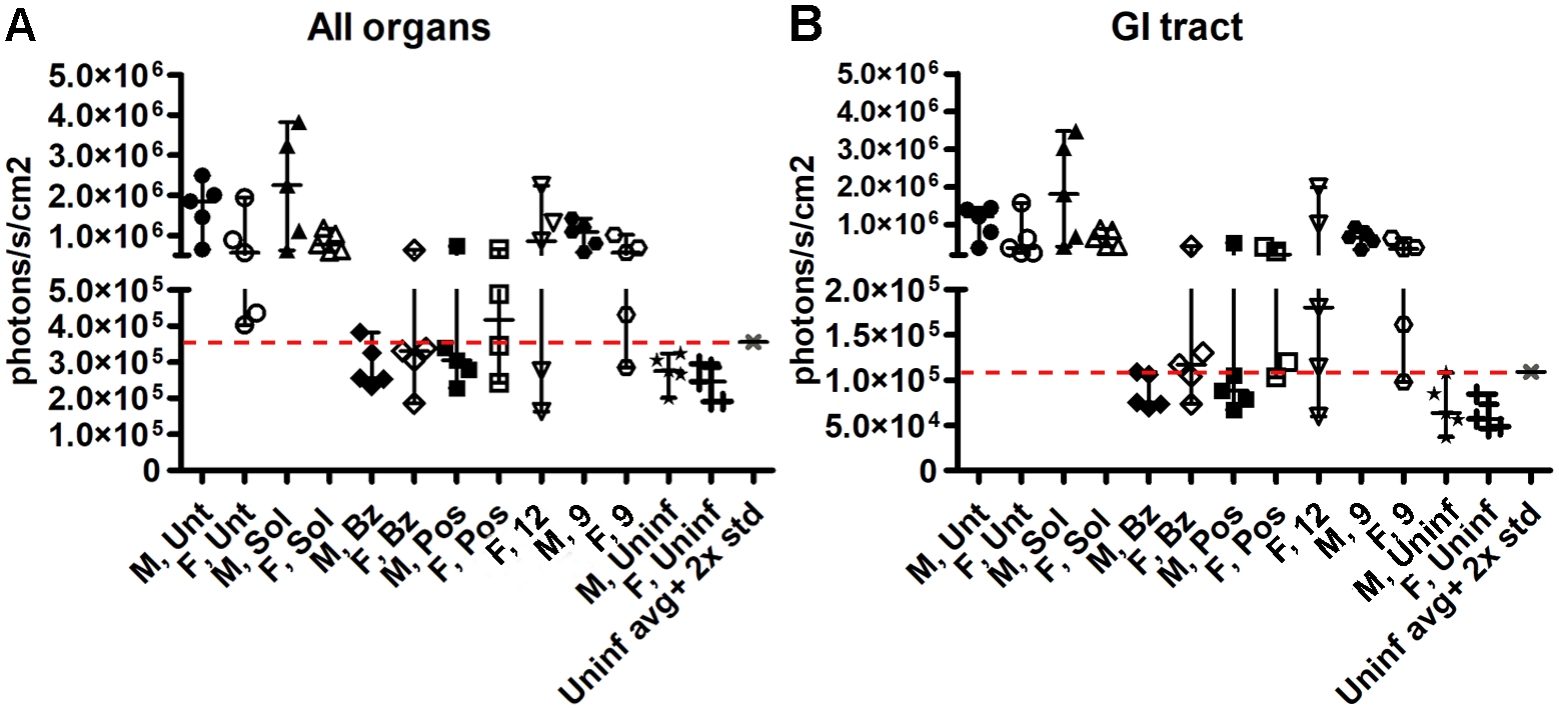
Quantitative analysis of *ex vivo* bioluminescence in the internal organs of chronically-infected mice. Each animal is represented by an individual data point. Treatment groups are labeled along the x-axis by the gender and drug name. (**A)** Cumulative signal from all the organs in each individual animal. (**B**) Signal from the GI tract alone. The majority of the bioluminescent parasites are associated with the GI tract.

*Ex vivo* bioluminescence imaging of internal organs was performed at 81 dpi. As previously described for this model [39,43], parasites were detected consistently in the gastro-intestinal (GI) tract in all *T*. *cruzi*-positive mice (Figs 7 and 8). Moreover, bioluminescence above the background level was also observed randomly in heart, skeletal muscles, liver and mesenteric fat in *T*. *cruzi*-positive mice throughout the groups, suggesting a dynamic infection process (Fig 7). *Ex vivo* quantification of the parasite burden in the organs analyzed (heart, liver, kidneys, lungs, spleen, gastro-intestinal tract, skeletal muscle and mesenteric fat) was consistent with the whole-mouse imaging. One female mouse treated with benznidazole showed traces of infection in the GI tract, while posaconazole-treated animals revealed *T*. *cruzi* in the GI tract and lung. For the experimental inhibitors, no parasites were detected in two females treated with **12** and one female treated with **9**. All other animals in the **9**- or **12**-treated groups showed *T*. *cruzi* bioluminescence in GI tract, liver, lung and mesenteric fat (Figs 7 and 8).

### Histopathological analysis

**Fig 9:**
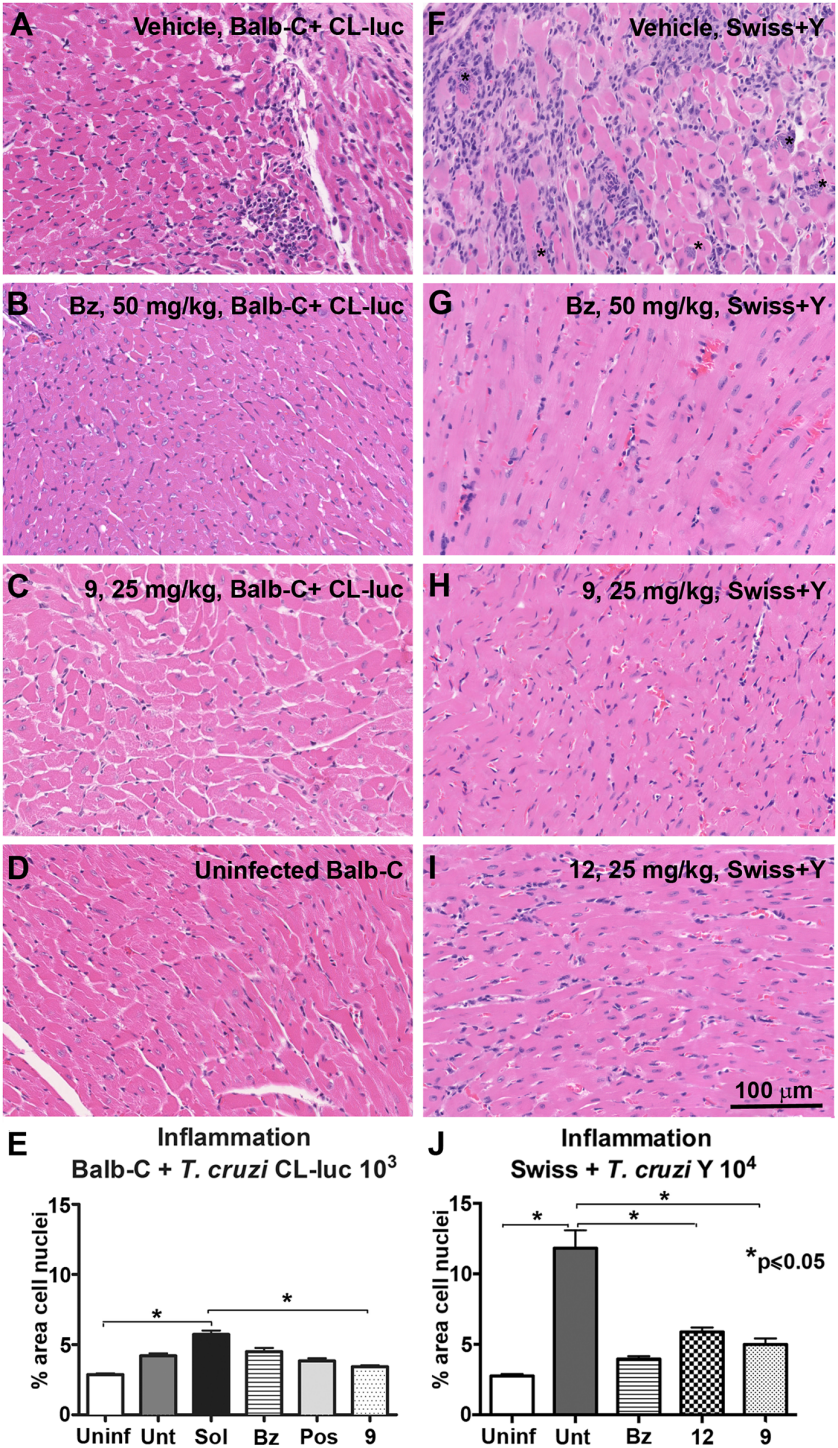
Histopathological analysis of heart tissue. H&E staining of heart tissue from acute models of *T*. *cruzi* infection. (**A-D**) BALB/c female mice infected with *T*. *cruzi* CL-luc. (**F-I**) Lethal infection of Swiss male mice infected with *T*. *cruzi* Y strain. In **F**, the parasite nests are labeled with the asterisks. Bar = 100μM. (**E, J**) Inflammation quantified using FIJI software.[44]. * Statistically significant by *t* test, p≤0.05.

Tissue architecture and inflammation in the heart was evaluated through conventional histology and H&E staining from both lethal acute and bioluminescent models, with the levels of inflammation being quantified using FIJI software [44]. Uninfected mice had normal cardiac tissue, as expected (Fig 9D). *T*. *cruzi* infection in untreated mice resulted in marked inflammation in heart tissue with inflammatory infiltrates and interstitial fibrosis in both acute models (Fig 9A and 9F). BALB/c mice infected with CL-luc showed mild cardiac inflammation when compared to Swiss mice infected with *T*. *cruzi* Y strain; the latter showed levels of inflammation at least 2× higher than the former and included the presence of amastigote nests, which were not observed in BALB/c mice infected with CL-luc (Fig 9A, 9F, 9E and 9J). In both models, mice treated with benznidazole (Fig 9B and 9G), **9** (Fig 9C and 9H), or **12** (Fig 9I) had normal heart tissue, with a significant reduction of inflammatory cells (Fig 9E and 9J) compared to vehicle treated controls, and no signs of interstitial fibrosis or amastigote nests, suggesting that the reduction of parasite load induced by treatment with the CYP51 inhibitors improved cardiac pathology, even without sterile cure.

### Effect of compounds in chronic infection

**Fig 10:**
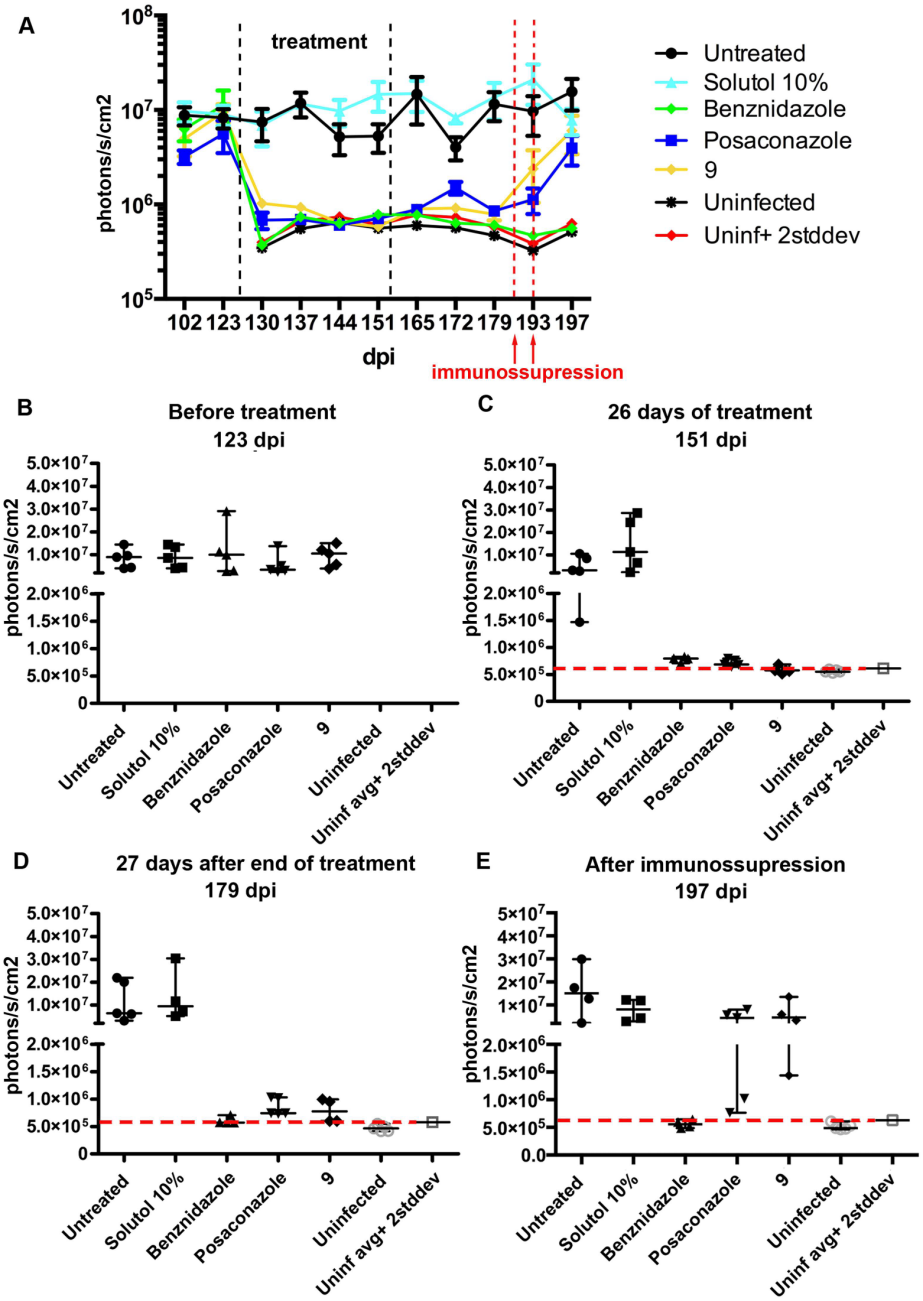
Effect of 4-aminopyridyl compounds in chronic infection. Parasite levels measured by bioluminescence detection in BALB/c males infected with *T*. *cruzi* CL-luc strain. (**A**) Average levels from all groups from 102 to 197 dpi. Individual values for each mouse in the groups represented separately on 123 dpi (**B**), 151 dpi (**C**), 179 dpi (**D**) and 197 dpi (**E**).

Most patients in need of treatment are in the chronic phase of Chagas disease. In this later stage, parasite load is low enough to require sensitive techniques for parasite detection [45]. To recapitulate these conditions, we evaluated performance of **9** in a chronic mouse model. Because males are more susceptible to infection than females, BALB/c males were infected with *T*. *cruzi* CL-luc strain allowing highly sensitive bioluminescence detection. Following the treatment scheme reported [46], the compounds were administered at 25 mg/kg for 28 days starting at 126 dpi, when chronic infection was established and the parasite signal was consistently detected. Similar to the acute model described above, *T*. *cruzi* bioluminescence levels dropped soon after the start of treatment. After 28 days, all treated groups, including **9**, showed only background luminescence (Fig 10A–10C). Mice were then followed for 4 weeks after compound administration had ceased. For up to 27 days post-treatment, groups treated with posaconazole or **9** had bioluminescence levels slightly above the background defined by the uninfected mice and 10–100× lower than the untreated controls (Fig 10D and 10E). Since the parasite load was below the detection level 4 weeks post-treatment, the animals were immunossupressed with cyclophosphamide. After 2 rounds of immunossupression, parasites relapsed as evidenced by the bioluminescence levels similar to those of the untreated controls.

## Discussion

A similarity in sterol biosynthesis pathways between *T*. *cruzi* and fungi is that both produce ergosterol and ergosterol-like sterols as membrane building blocks [47]. This similarity encouraged the application of antifungal drugs for the treatment of Chagas disease. However, in human clinical trials for Chagas disease, both posaconazole and ravuconazole failed to demonstrate superiority to the current standard-of-care drug, benznidazole, using PCR as a marker of continued or reactivated *T*.*cruzi* infection [25,27]. The failure of posaconazole and ravuconazole to attain sterile cure in humans raised concerns about the CYP51 target. Differential activity of CYP51 inhibitors against the replicative (amastigote) and non-replicative (trypomastigote) stages of *T*. *cruzi*, a slow-acting mechanism of action, and the stochastic nature of *T*. *cruzi* infection with the non-replicating or rarely-replicating cryptic amastigotes ‘hidden’ inside the tissues [39], were listed as potential drawbacks of the CYP51 target [28,48]. On the other hand, it has been argued that both the dose and the duration of anti-fungal agents used in the clinical trials to treat human *T*. *cruzi* infection have been suboptimal. Urbina et al. noted that the plasma exposure in patients for the dose used in clinical trials corresponds to 10–20% of the curative dose in mice [29,49]. The post-clinical trial tendency to balance risks in the Chagas drug discovery portfolio, and to identify drug candidates aimed at other molecular targets is logical. At the same time, it is critical to not reject promising targets based on clinical studies with drugs not properly optimized, dosed, or clinically evaluated.

An important, but overlooked factor, that may have affected the performance of the anti-fungal drugs in Chagasic patients, is the loose drug-target fit demonstrated by posaconazole in co-crystal structures with *Trypanosome* CYP51. The electron density of the bound drug is poorly defined and its pendant phenyl-2-hydroxy-pentantriazolone group adopts alternative conformations to make multiple interactions outside of the active site [50,51]. Several novel CYP51 inhibitors developed in this collaboration [33–37] and elsewhere [51–53] demonstrated drug-target fits superior to posaconazole. The most potent 4-aminopyridyl-based inhibitors of the 4-aminopyridyl-based series bind entirely in the CYP51 target interior, making tight interactions with hydrophobic residues constituting the CYP51 active site [35,37]. Improved drug-target interactions may be responsible, at least in part, for the superior potency of the experimental inhibitors in the acute and chronic mouse models of infection [54].

Neither of the two lead compounds of the 4-aminopyridyl-based series evaluated in these studies attained a sterile cure. However, both leads were proven safe for long term administration in mice and, efficiently suppressed parasitemia in both acute and chronic models of infection. In the acute phase, compounds improved survival in highly stringent acute mouse models, protected mice from hepatic injury, and drastically reduced acute cardiac inflammation. In a model of chronic Chagas disease, **9** prevented spontaneous *T*. *cruzi* relapse for up to 4 weeks post-treatment. Similar results—supression of parasitemia, no spontaneous relapse after treatment withdrawal and parasite reactivation after immunossupression—were also achieved by other research groups using CYP51 inhibitors based on different molecular scaffolds [41,46,55].

Collectively, **9** is more efficacious in the treatment of the chronic phase of the disease with low parasite load. Although **9** did not eradicate cryptic reservoirs of parasites *in vivo* after 28 days of treatment, it successfully kept parasites under control and prevented the inflammation responsible, in part, for cardiac tissue damage. This outcome is not unique for inhibitors that target the ergosterol synthesis pathway. Treatment of chronically infected mice with N,N-dimethylsphingosine, an inhibitor of sphingosine kinase, also failed to produce a sterile cure, but reduced parasite load leading to a marked decrease in inflammation and fibrosis. Furthermore, there was a reduction of inflammatory mediators and an improvement of heart function measured as exercise capacity [56]. In addition, mice infected with resistant strains of *T*. *cruzi* showed decreased tissue parasitemia, reduced myocarditis and less electrocardiographical alterations after treatment with benznidazole, even though the drug failed to completely eliminate parasites in this model [57].

Several mechanisms have been proposed to explain the pathogenesis of Chagas’ cardiomyopathy, including parasite-dependent inflammation, autoimmunity, autonomic neuronal degeneration and damage of microvasculature [58,59]. Although more than one mechanism may be involved in Chagas disease pathogenesis, a consensus is that tissue damage is related to parasite persistence [58–60]. At the same time, 60–70% of infected individuals are asymptomatic. A balance between host and parasite in asymptomatic cases may be maintained by expression of the anti-inflammatory cytokine IL-10, while cardiomyopathy is associated with inflammation triggered by IFN-gamma and TNF-alpha [61]. Reducing the parasite burden diminishes inflammation even without complete elimination of the parasite [60]. In this regard, several non-randomized clinical trials have shown that etiological treatment of chronic patients with benznidazole resulted in slower progression to advanced stages of cardiomyopathy evaluated by electrocardiography and echocardiography [17,62].

Sterile cure is a highly desirable treatment outcome, however, it may not always be achieved, and drug discovery efforts are often hampered by deficiencies in understanding the nuances of disease pathogenesis. There is currently no sterile cure of HIV infection. The desirable outcome of the antiretroviral treatment is a long term plasma HIV-RNA count below 50 copies/ml [63]. The WHO recommends antiretrovirals in people of all ages, including pregnant women as soon as the diagnosis is made; once treatment is begun, it is recommended to continue throughout the entire life span without interruptions [63]. Benefits of treatment include a decreased risk of progression to AIDS and a decreased risk of death [64]. Highly active antiviral therapy options are available as drug ‘cocktails’ consisting of at least three medications belonging to at least two different classes of antiviral agents [65]. As of 2017, 19.5 million people are accessing antiretroviral therapy and more than half of all people living with HIV are on treatment [66].

By analogy with HIV/AIDS, the treatment option of non-toxic medications should be developed for Chagas patients to slow down progression to cardiomyopathy and to improve life expectancy and quality of life. With the scarce arsenal of anti-*T*. *cruzi* agents, the drug discovery community cannot afford to be prejudiced against CYP51, or any other target, if the inhibitors have acceptable safety profiles and achieve a marked reduction in parasite load, even in the absence of sterile cure. Regardless of the molecular target affected by the drug, development of an efficacious and safe treatment for Chagas disease would be a breakthrough for society, medicine and science.

## Material and methods

### Ethics statement

Research performed at UC San Diego was conducted in compliance with the Animal Welfare Act and other federal statutes and regulations relating to animals and experiments involving animals and adheres to the principles stated in the Guide for the Care and Use of Laboratory Animals, National Research Council, 2011. The facility where this research was conducted is fully accredited by the Association for Assessment and Accreditation of Laboratory Animal Care International. Animal research was conducted under approved protocol S14187 from the Institutional Animal Care and Use Committee, University of California, San Diego. Research performed at Oswaldo Cruz Foundation—FIOCRUZ, Rio de Janeiro, Brazil, was approved by the Committee for Ethics in the Use of Animals of FIOCRUZ, under protocol number LW-37/13 and is in compliance with Brazilian Federal Law number 11794/08, Federal Brazilian Decree number 6899/09 and Brazilian Normative Resolution number 1 (July 9th, 2010) of the National Council for the Control of Animal Experimentation. Euthanasia was accomplished by CO_2_ inhalation or by sodium pentobarbital overdose (60 mg/kg), followed by cervical dislocation. These methods of euthanasia have been selected because they cause minimal pain and distress to animals, are relatively quick, and do not adversely impact interpretation of the results of studies. All methods are in accord with the recommendations of the Panel on Euthanasia of the American Veterinary Medical Association.

### Materials

Compounds **9** and **12** were synthesized by following the procedures previously reported [35,37].

### Animals

*In vivo* experiments were performed at the University of California San Diego (UCSD), La Jolla, California, USA and Oswaldo Cruz Foundation—FIOCRUZ, Rio de Janeiro, Brazil. At FIOCRUZ, Swiss Webster male and female mice weighting 18–20 g were obtained from CEMIB (Centro Multidisciplinar para Investigação Biológica), UNICAMP (Campinas, SP, Brazil). At UCSD, male and female 6 weeks old BALB/c mice, in the same weight range, were purchased from Jackson Laboratories (Farmington, CT, USA). Mice were housed in a maximum number of 5 animals per cage and kept in a conventional room at 20 to 24°C under a 12 h/12 h light/dark cycle. The animals were provided with sterilized water and chow ad libitum.

### Toxicity assays

Acute toxicity was evaluated by administration of escalating doses of the compounds to male and female Swiss Webster mice (n = 2/group) orally by gavage, at 100 ul/hour 50 mg/kg dose formulated in 20% solutol (also known as Kolliphor HS15) (Sigma #42966). The general health of the animals was closely monitored for up to 48 h and the last dose before the onset of toxic symptoms were observed was defined as NOAEL according to the OECD guidelines. Cumulative toxicity after prolonged treatment was evaluated using BALB/c females (n = 5/group) treated orally by gavage with the experimental CYP51 inhibitors, **9** (25 mg/kg) or **12** (25 mg/kg), dissolved in 10% solutol, at 100 ul/dose, b.i.d, for 28 consecutive days. Mice were weighed once a week and their general health was assessed daily. After treatment, mice were euthanized, blood was collected for analysis of several biochemical markers of general health. Brain, heart, liver, kidney, gastro-intestinal (GI) tract and lungs were collected, briefly rinsed in PBS and fixed in buffered formalin solution including 10% formaldehyde, 33 mM NaH_2_PO_4_, 45 mM Na_2_HPO_4_ for histological evaluation.

### Parasites

The *T*. *cruzi* Y parasites were obtained from the bloodstream of infected Swiss Webster mice at the peak of parasitemia, as previously described [67]. Transgenic *T*. *cruzi* CL Brener parasites expressing a red-shifted luciferase that emits light in the tissue-penetrating orange-red region of the spectrum (a gift from Dr. John Kelly, London School of Hygiene and Tropical Medicine, London, United Kingdom), were obtained as described previously [39]. Epimastigote forms were maintained at 28°C in LIT media supplemented with 10% FBS and 100 μg/ml of antibiotic G418 to keep selective pressure in favor of the luciferase marker [68]. Epimastigotes were induced to differentiate to trypomastigotes through metacyclogenesis as previously described [69]. Metacyclic trypomastigotes were used to infect C2C12 myoblasts monolayers. After 5–7 days, trypomastigotes released in supernatant were collected by centrifugation for 15 min at 3300 rpm, re-suspended in DMEM and used to infect mice.

### Infection of mice

Swiss Webster male mice weighting 18–20 g were infected intraperitoneally with 10^4^ bloodstream trypomastigote form of *T*. *cruzi* Y parasites. For bioluminescence imaging, six week old BALB/c male and female mice were infected by intraperitoneal injection with 10^3^ *T*. *cruzi* CL-luc trypomastigotes derived from cell culture supernatant.

### Treatment strategies

All drugs were solubilized in 10% solutol and administered orally, b.i.d, at previously optimized doses: 25 mg/kg for **9** and **12**, 50 mg/kg for benznidazole, and 20 mg/kg for posaconazole. The treatment of Swiss mice acutely infected with *T*. *cruzi* Y strain was started with parasitemia onset at 5 days post-infection (dpi). The treatment of BALB/c mice infected with CL-luc parasites started at 14 dpi (acute phase), when parasitemia reached a peak as detected by bioluminescence, or at 126 dpi (chronic phase), when a chronic state of infection was established [46]. In all models, only parasite-positive mice (5 mice/group) were used in the treatment course lasting for 28 days. To assess if sterile cure was achieved, immunossupression was performed in the chronic model of infection 4 weeks after the end of treatment, with two doses of cyclophosphamide (200 mg/kg) by intraperitoneal (i.p.) injection at 3-day intervals.

### Parasitemia determination

In Swiss mice acutely infected with *T*. *cruzi* Y strain, parasites in the blood of each animal were quantified by using the Pizzi-Brener method. The total number of parasites are counted in 50 fields under 400X magnification of freshly prepared blood samples (5 μl drops) obtained from the tail veins of mice, collected 3 times a week, starting at 5 dpi and continued until the end of treatment [70]. Mortality was monitored daily and % survival was calculated using GraphPad prism software.

### Bioluminescent imaging

BALB/c mice infected with parasites carrying a bioluminescent marker were imaged at 13 dpi, before treatment was initiated, and then once a week, both during the 28-day treatment period and 39 days post-treatment, as previously described [35]. Briefly, mice were injected i.p. with 150 mg/kg D-luciferin potassium salt in PBS (Gold Biotechnology, St. Louis, MO), and 5 minutes later, anesthetized by isofluorane inhalation (3–5%) and imaged using IVIS Lumina *in vivo* imaging system (PerkinElmer, Waltham, MA) with 180s exposure time. Data acquisition and analysis were performed with the LivingImage V4.1 software (PerkinElmer, Waltham, MA). Uninfected controls were imaged in parallel to establish a negative threshold.

### Ex-vivo imaging

To evaluate sites of parasite persistence in BALB/c mice infected with *T*. *cruzi* expressing luciferase, we performed *ex vivo* imaging of selected internal organs according to the protocol adapted from Lewis et al., 2014 [39]. Briefly, the animals were injected i.p. with 150 mg/kg of D-luciferin, euthanized in a CO_2_ chamber and perfused with 10 ml of D-luciferin. Then, heart, liver, kidneys, lungs, spleen, mesenteric fat, skeletal muscle (excised from left thigh) and the whole gastro-intestinal (GI) tract were removed, placed in a petri dish with PBS containing 300 μg/ml of D-luciferin, and imaged using the IVIS Lumina system.

### Histology

Brain, heart, liver, kidney, GI tract and lungs from uninfected mice for toxicity analysis, and heart and GI tract from infected animals were removed and fixed as described above. Samples were processed for routine histologic examination in the Histology Core of Moore Cancer Center (UCSD), embedded in paraffin, sectioned and stained with hematoxylin and eosin. The slides were scanned using Nanozoomer Slide Scanner (Hamamatsu Photonics, NJ, USA) and images were obtained through NDP viewer software (Hamamatsu Photonics, NJ, USA).

### Histopathology analysis

To quantify levels of inflammation, 5 random images of mouse hearts (10× magnification) were obtained from each animal, 5 animals/group. At this magnification, 5 fields comprise the majority of the area of the heart section. Image processing was performed using Fiji software [44], where cell nuclei was segmented through the Particle Analyzer plugin and the fraction of total area of the image occupied by all cell nuclei was then measured. Even though cardiomyocytes and cardiac fibroblasts cell nuclei are being counted together with inflammatory cells, uninfected heart sections were used as controls and provide a baseline number.

### Blood analysis

Terminal blood collection was performed via cardiac cavity exsanguination in uninfected and *T*. *cruzi*-infected mice. Blood was collected in serum separator tubes (Microtainer, BD Biosciences), allowed to clot for 0.5–2.0 h and then centrifuged for 90 s at 10000 g. Serum was removed and analyzed at the Central Animal Facilities of the Oswaldo Cruz Foundation (Rio de Janeiro, Brazil, CECAL/Fiocruz platform) using Vitros 250 (Ortho Clinical-Johnson & Johnson), or at the UC Davis Comparative Pathology Laboratory (Davis, CA, USA), where samples were analyzed using Roche Cobas Integra 400 Plus clinical chemistry analyzer. In both facilities, tests were performed for electrolytes and enzyme metabolites indicative of liver, kidney and cardiac functions, including alanine aminotransferase (ALT), aspartate aminotransferase (AST), total bilirubin (BIL), albumin (ALB), alkaline phosphatase (ALP), blood urea nitrogen (BUN), creatinine (CRE), urea, calcium, phosphorus, glucose, total protein.

### Statistical analysis

Student’s *t*-test was used for evaluation of differences in experimental data between groups. Values were considered statistically significant when p≤ 0.05. Statistics were analyzed by GraphPad Prism Software.
